# Benchmark study of feature selection strategies for multi-omics data

**DOI:** 10.1186/s12859-022-04962-x

**Published:** 2022-10-05

**Authors:** Yingxia Li, Ulrich Mansmann, Shangming Du, Roman Hornung

**Affiliations:** Institute for Medical Information Processing, Biometry and Epidemiology, University of Munich, Marchioninistr. 15, 81377 Munich, Germany

**Keywords:** Multi-omics data, TCGA, Benchmark, Feature selection, Classification

## Abstract

**Background:**

In the last few years, multi-omics data, that is, datasets containing different types of high-dimensional molecular variables for the same samples, have become increasingly available. To date, several comparison studies focused on feature selection methods for omics data, but to our knowledge, none compared these methods for the special case of multi-omics data. Given that these data have specific structures that differentiate them from single-omics data, it is unclear whether different feature selection strategies may be optimal for such data. In this paper, using 15 cancer multi-omics datasets we compared four filter methods, two embedded methods, and two wrapper methods with respect to their performance in the prediction of a binary outcome in several situations that may affect the prediction results. As classifiers, we used support vector machines and random forests. The methods were compared using repeated fivefold cross-validation. The accuracy, the AUC, and the Brier score served as performance metrics.

**Results:**

The results suggested that, first, the chosen number of selected features affects the predictive performance for many feature selection methods but not all. Second, whether the features were selected by data type or from all data types concurrently did not considerably affect the predictive performance, but for some methods, concurrent selection took more time. Third, regardless of which performance measure was considered, the feature selection methods mRMR, the permutation importance of random forests, and the Lasso tended to outperform the other considered methods. Here, mRMR and the permutation importance of random forests already delivered strong predictive performance when considering only a few selected features. Finally, the wrapper methods were computationally much more expensive than the filter and embedded methods.

**Conclusions:**

We recommend the permutation importance of random forests and the filter method mRMR for feature selection using multi-omics data, where, however, mRMR is considerably more computationally costly.

**Supplementary Information:**

The online version contains supplementary material available at 10.1186/s12859-022-04962-x.

## Background

In the past few years, various types of omics data have become available on The Cancer Genome Atlas (TCGA) [1] such as data on genomics, epigenomics, transcriptomics, proteomics, metabolomics and microbiomics. It is well-known that a large amount of omics data is not informative for prediction because they are either redundant or irrelevant [2, 3]. The importance of feature selection is beyond doubt and different methods have been developed to deal with high-dimensional data. However, it is unclear, how feature selection should be performed for multi-omics data, that is, data for which there are measurements of several types of omics data from the same patients. This is because the predictive information in the different omics data types is overlapping, the amount of predictive information varies between the data types, and there are interactions between features from different data types [4, 5].

Using different types of omics data effectively is challenging. An important characteristic of multi-omics data is the large dimensionality of the datasets. To address the issue of the large number of input features, feature selection algorithms have become crucial components of the learning process. The feature selection process aims to detect the relevant features and discard the irrelevant ones. Successful feature selection can lead to an improvement of the inductive learner, either in terms of learning speed, generalization capacity, or simplicity of the induced model. In addition, the specific structure of multi-omics data may be accounted for when selecting feature subsets. Lastly, apart from multi-omics data, in most cases the corresponding phenotypic dataset features several clinical covariates. Several studies have demonstrated that combining omics data with clinical data improves predictive performance [6, 7]. Therefore, clinical variables should be considered as well.

Presently, numerous feature selection methods exist which can be classified into different types according to specific principles [8]. For example, based on the relationship between the feature selection step and the learning procedure of the prediction rule, they can be classified as filter, wrapper, embedded, or hybrid methods. Based on the type of feature selection output, they can be divided into feature rank and subset selection methods. Some studies have compared feature selection methods for single-omics data, however, these studies often had limited scopes and no sufficiently large-scale systematic comparison in the context of multi-omics data has been conducted. A pioneering study by Abusamra [9] analyzed the performance of eight different filter-based feature selection methods and three classification methods, using only gene expression data of glioma. Liu et al. [10] conducted a comparative study of five feature selection methods using two datasets (Leukemia and Ovarian cancer). A study by Verónica et al. [11] investigated 11 feature selection methods using 11 datasets, including seven filter methods, two embedded methods, and two wrapper methods, but this analysis was based on synthetic data. Though many studies have investigated the strengths and weaknesses of existing feature selection algorithms [12–14], the choice of the most appropriate approach for a given task remains difficult [15].

In this paper, we aim to fill this gap for multi-omics data by providing a large-scale benchmark experiment comparing different feature selection methods and strategies. It is based on 15 cancer datasets from TCGA and focuses on classification. We compared four filter methods, two embedded methods, and two wrapper methods with respect to their performance in selecting combinations of features that perform well in classification. Where the output type was feature rank, we explored the effect of using different numbers of selected features on the classification performance. We also investigated the impact of performing the feature selection separately for each data type and concurrently for all data types at the same time. Finally, we studied the impact of combining multi-omics data with clinical data on the classification performance.

## Results

In the following, the results of the benchmark study will be presented. Detailed descriptions of the design of this study and the compared featured selection methods are given in “Methods” section. Consulting the latter section before reading the current section should make it easier to follow the results presented in the following.

### Main findings

We discuss the results obtained for the area under the receiver operating characteristic curve (AUC) here, but analogous conclusions can be drawn for the accuracy and the Brier score (Brier). The results obtained for the latter two measures are shown in Additional file 1: Figures S1–S4.

**Fig. 1:**
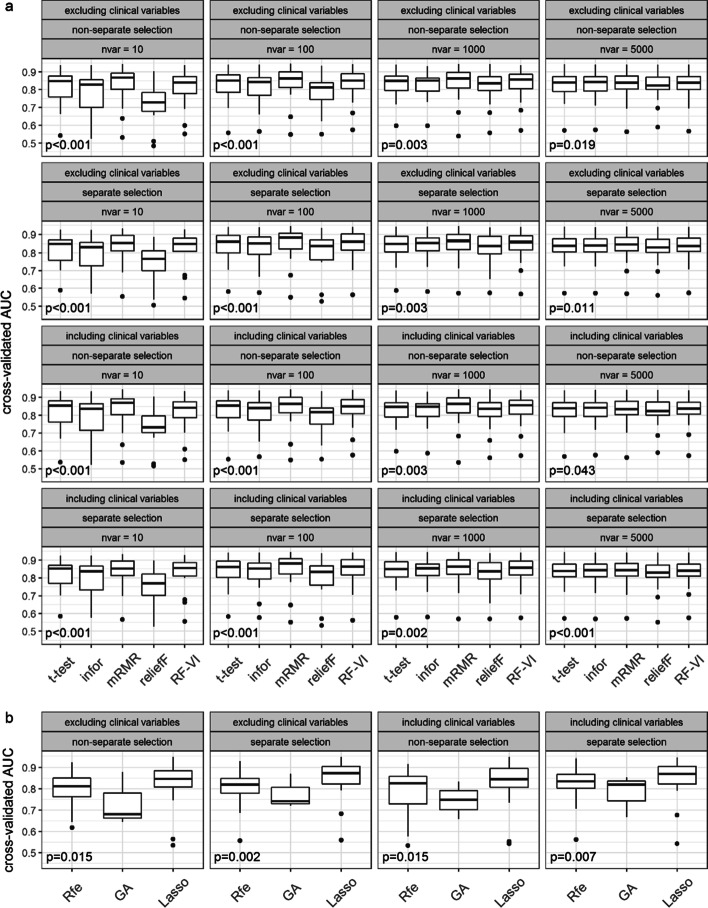
Prediction performance using RF after feature selection. Panels **a** and **b** show the distributions of the mean cross-validated AUC values across the datasets for all rank and subset evaluation methods, respectively. The *p-*values show the results of the Friedman tests

**Fig. 2:**
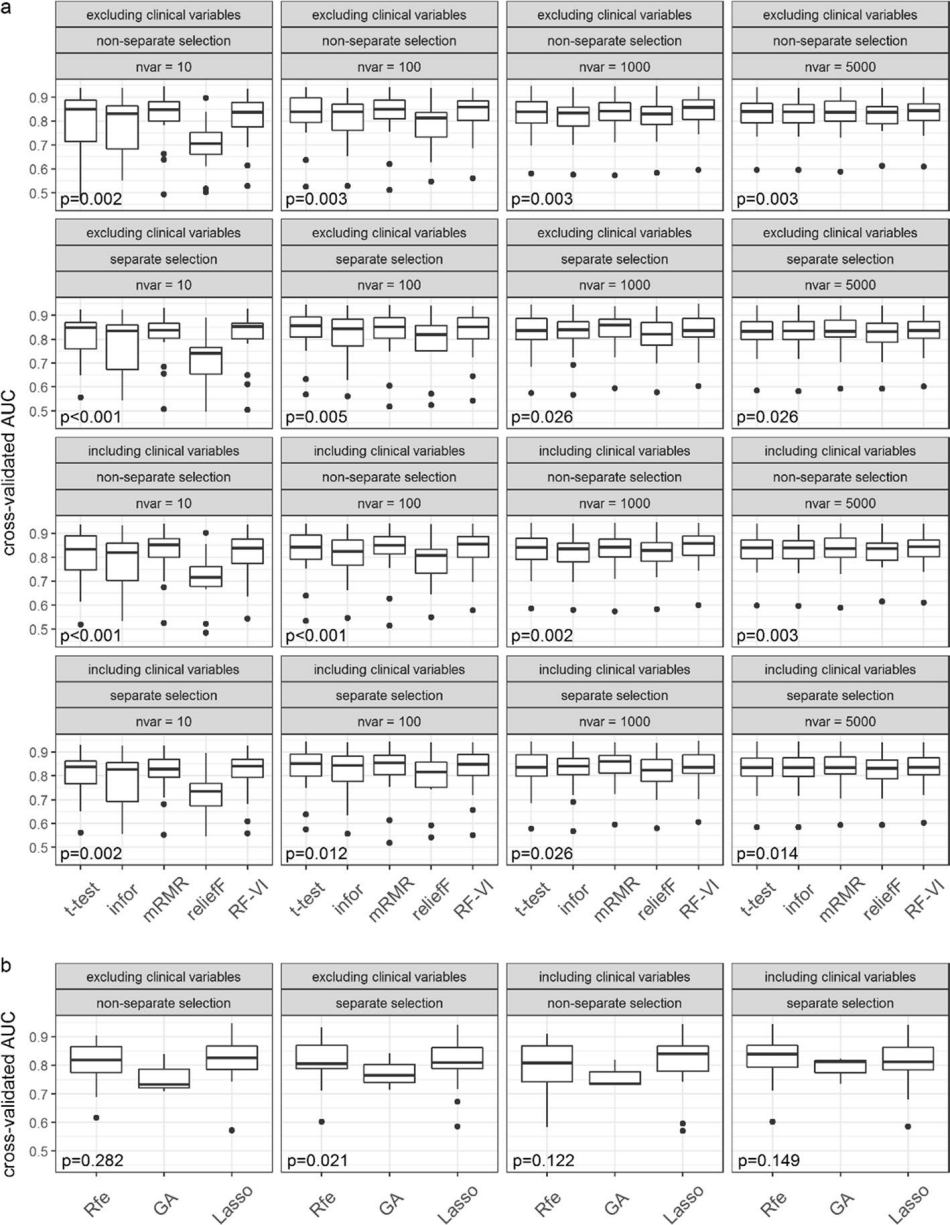
Prediction performance using SVM after feature selection. Panels **a** and **b** show the distributions of the mean cross-validated AUC values across the datasets for all rank and subset evaluation methods, respectively. The *p-*values show the results of the Friedman tests

Figures 1 and 2 show the results obtained for random forests (RF) and support vector machine (SVM), respectively. Figures 1a and 2a show the distributions of the mean cross-validated AUC values across the datasets for all rank methods. Figures 1b and 2b show the mean cross-validated AUC values obtained for all subset evaluation methods.

As seen in Figs. 1a and 2a, with regards to the AUC of SVM and RF, the performance of the rank methods varies strongly between different nvar values. For nvar = 10, there are strong differences in performance between the methods and for the worst methods, there is also a considerable variability across datasets. For all ranking methods, these differences get smaller for larger nvar values, and starting with nvar = 1000, all methods performed similarly well. On average, the Minimum Redundancy Maximum Relevance method (mRMR) and the permutation importance of random forests (RF-VI) performed best among all methods. These methods already performed well for nvar = 10, meaning that these methods can also be used to construct classifiers using few features and there is no need to consider larger numbers of features. For both classification methods, reliefF performed much worse for small nvar values. For RF, information gain (infor) also had a much weaker performance in this range of nvar values.

The genetic algorithm (GA) performed worst among the subset evaluation methods for both classification methods. The least absolute shrinkage and selection operator (Lasso) performed best for RF and comparable to recursive feature elimination (Rfe) for SVM. For RF, Lasso performed best among all methods, but the improvement in performance over the other best-performing methods was not strong. Moreover, with an average of 190 selected features, Lasso required more features than mRMR and RF-VI. The wrapper methods Rfe and GA selected 4801 and 2755 features on average, respectively.

Including the clinical information did not improve the predictive performance. However, we did not prioritize the clinical information in the feature selection and in the classification, which likely explains this result. Previous work has demonstrated that prioritizing the clinical information in the prediction can considerably improve the performance [6, 16].

There were also no notable differences between results when performing the feature selection for all data types simultaneously or separately. There was one exception in the case of RF: Here, performing the selection separately for each data type delivered better prediction results in the case of GA.

The results of the Friedman test for performance differences between the methods were significant with the exception of the results for SVM in the case of the subset evaluation methods. Here, we saw significant differences only for separate selection when excluding the clinical variables. There were less significant results in the case of the other two performance metrics (Additional file 1: Figures S1–S4). In the case of the accuracy, for RF, the results were all significant with the exception of the rank methods for the settings with the largest nvar value 5000. However, for the SVM there were frequent non-significant results; in the case of the rank methods, in particular for larger numbers of nvar.

### The best performing methods per setting

In Table 1, for each setting, the feature selection strategies that performed best with respect to the AUC (averaged across all datasets) are displayed for the results obtained with RF. As seen in Table 1, mRMR performed best among the rank methods regardless of the setting, while Lasso outperformed the other two subset evaluation methods for all settings; mRMR achieved its best AUC values for nvar = 100 and separate selection. Lasso also performed best with respect to the AUC for separate selection. In general, the results did not notably differ between excluding and including the clinical features for any of the settings. The corresponding results for SVM are shown in Additional file 1: Table S1. Here, while mRMR was still the best-performing method most frequently, there was a greater variety in the best-performing methods. For example, RF-VI performed best frequently as well and Rfe was the best method in two of the four settings for the subset evaluation methods.

**Table 1:** The best performing methods (according to the AUC) per setting

nvar	selsep	clivar	Selector	AUC	Brier	accuracy
10	Yes	Yes	mRMR	0.8299	0.1347	0.8217
10	Yes	No	mRMR	0.8266	0.1357	0.8189
10	No	Yes	mRMR	0.8263	0.1323	0.8281
10	No	No	mRMR	0.8247	0.1331	0.8261
100	Yes	Yes	mRMR	0.8405	0.1287	0.8359
100	Yes	No	mRMR	0.8406	0.1286	0.8363
100	No	Yes	mRMR	0.8345	0.1307	0.8311
100	No	No	mRMR	0.8354	0.1307	0.8290
1000	Yes	Yes	mRMR	0.8374	0.1342	0.8196
1000	Yes	No	mRMR	0.8376	0.1339	0.8200
1000	no	yes	mRMR	0.8290	0.1364	0.8171
1000	No	No	mRMR	0.8274	0.1366	0.8172
5000	Yes	Yes	mRMR	0.8264	0.1383	0.8148
5000	Yes	No	mRMR	0.8260	0.1384	0.8128
5000	No	Yes	mRMR	0.8227	0.1401	0.8111
5000	No	No	mRMR	0.8215	0.1402	0.8107
-	Yes	Yes	Lasso	0.8387	0.1335	0.8219
-	Yes	No	Lasso	0.8413	0.1330	0.8219
-	No	Yes	Lasso	0.8190	0.1374	0.8205
-	No	No	Lasso	0.8185	0.1386	0.8213

The values of the performance metrics were obtained by averaging over the cross-validation repetitions and datasets; ‘nvar’ denotes the number of selected features, ‘selsep’ whether the features were selected separately by data type, and ‘clivar’ whether clinical variables were included or not

### The best performing methods and settings per dataset

For each dataset, the results obtained with RF for the best methods and settings according to the AUC are displayed in Table 2. For most datasets, the best performance was achieved with the mRMR selector, while Lasso performed best second most often. For all datasets where the mRMR selector performed best, it used only a small subset of features (nvar = 10 or 100). For no dataset, the *t*-test or reliefF were able to achieve the best classification results. For SVM (Additional file 1: Table S2) *t*-test and RF-VI performed best most often, followed by mRMR and Lasso. Another difference to the results obtained with RF is that the best settings more often featured large nvar values.

**Table 2:** The best performing methods and settings (according to the AUC) per dataset

Dat	Selector	nvar	selsep	clivar
BLCA	mRMR	100	Yes	Yes
BRCA	Lasso	–	No	Yes
COAD	mRMR	10	No	Yes
ESCA	infor	1000	No	No
HNSC	mRMR	10	Yes	Yes
LGG	Lasso	–	No	No
LIHC	mRMR	100	Yes	Yes
LUAD	mRMR	100	No	No
LUSC	Rfe	–	No	No
PAAD	mRMR	10	Yes	Yes
PRAD	mRMR	100	yes	Yes
SARC	GA	–	Yes	Yes
SKCM	mRMR	100	No	No
STAD	RF-VI	100	Yes	No
UCEC	Lasso	–	Yes	No

Here, ‘nvar’ denotes the number of selected features, ‘selsep’ whether the features were selected separately by data type, and ‘clivar’ whether clinical variables were included or not

### Computation time

The time it takes to run a method is an important factor that influences its applicability. For this reason, we not only evaluated the performance of the feature selection methods with respect to the performance of the resulting prediction rules, but also in terms of computation time. The latter was measured as the time needed for one feature selection process to be completed on the training data. All values were obtained by averaging over the cross-validation repetitions and then averaging across the datasets.

**Fig. 3:**
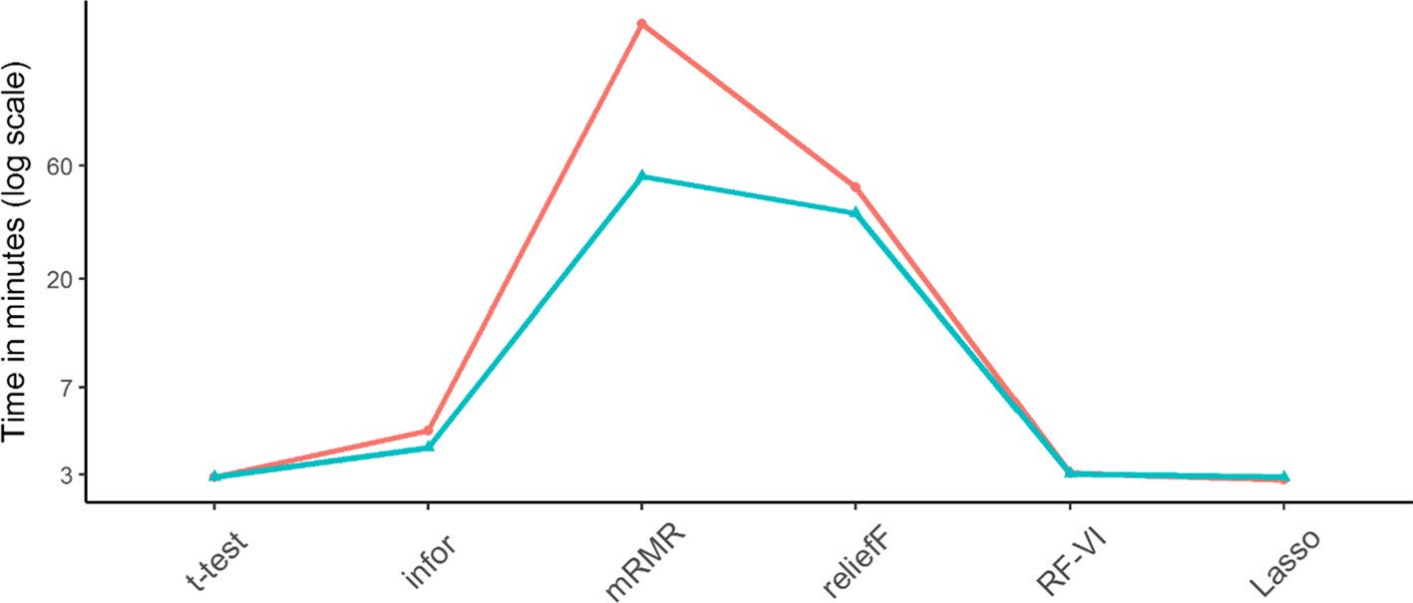
Mean computation times of feature selection methods averaged across the different datasets. The red and the blue lines indicate the results obtained when selecting from all data types concurrently and separately, respectively

Since the wrapper methods take considerably more time (Rfe: more than 1 days, GA: more than 2 days), Fig. 3 only shows the computation times of the filter and embedded methods. For the rank methods, we considered the feature selection times resulting when setting nvar = 5000. As seen in Fig. 3, with concurrent selection, mRMR and reliefF took the longest, but the computation times of mRMR were reduced for separate selection. Finally, *t*-test, RF-VI, and Lasso took about the same time regardless of whether the selection was performed separately from each data type or concurrently from all data types.

**Fig. 4:**
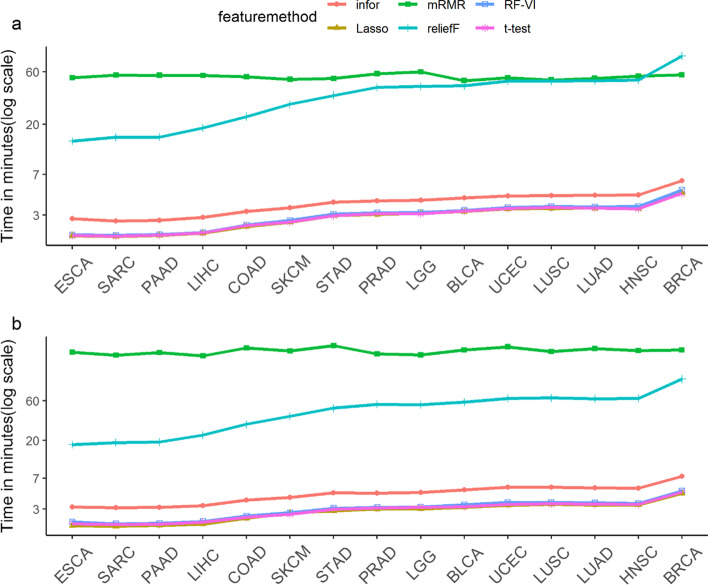
Mean computation times of feature selection methods for the different datasets. Panel **a** shows the results obtained for separate selection and panel **b**, those obtained for concurrent selection from all data types

Of course, the computation times may also depend on the size of the dataset. Figure 4 shows the average computation times of one feature selection process for the different selectors and datasets. The datasets are ordered from smallest (ESCA) to largest (BRCA) with respect to the numbers of values in the datasets. Except for in the case of reliefF, the computation times increased only slightly for larger datasets.

## Discussion

For the ranking methods infor and reliefF, the number of selected features strongly affected the predictive performance in our benchmark study. If the number of selected features was small, these methods performed considerably worse. However, the ranking methods mRMR and RF-VI were observed to be quite robust with respect to the nvar value, where the predictive performance was already strong for small nvar values. These methods can thus be used for selecting features for both prediction rules based on few genes and prediction rules based on many genes. Using Lasso for feature selection was also associated with strong predictive performance, but the number of selected features was quite large.

We did not observe notable differences in predictive performance when selecting the features concurrently from all data types or separately for each data type. However, from a theoretical point of view, we would assume that separate selection may degrade the performance for wrapper methods as this type of selection may lead to redundant feature subsets from different data types. We did, however, not observe a degraded performance when using separate selection for the wrapper methods.

In our study, including clinical variables did not improve the predictive performance. However, it must be considered that the clinical variables are very small in number compared to the omics features. As noted above, we did not prioritize the clinical variables over the omics features. It can be highly beneficial to take the clinical variables into account because they contain important predictive information, but they need to be prioritized over the omics variables to exploit this predictive information [6, 16].

The choice for a method is not only influenced by its performance, but also by the computation time associated with it. Among the best-performing methods RF-VI and Lasso required reasonable computation times. However, mRMR took excessively long, in particular for concurrent selection. In general, Rfe and GA do not seem to be suitable for multi-omics data because the computation times associated with these methods were much too long for practical use in our benchmark study.

As seen in Figs. 1 and 2, there is strong variability of the results across different datasets. The superiority of one method over another is dependent on the dataset under consideration. This emphasizes the importance of large benchmark studies which use many datasets, like the one performed in this paper. The fact that we need many observations because of the high variability among them is well known to statisticians when performing sample size calculations, but is often ignored when designing benchmark experiments using real datasets [17]. Had we conducted the study with four, six, or eight datasets (as is common in the literature), we would have obtained different and more unstable results. The variability of results across datasets also illustrates that no method is preferable over all other methods for all datasets. With benchmark studies, we always only measure mean performances but the rankings of the performances of the methods vary across datasets. The fact that, in the case of the accuracy and the Brier score, we did not observe statistically significant differences between the methods for many of the settings confirms the conclusion of Boulesteix et al. [17] that large numbers of datasets are required to obtain significant differences in benchmark studies.

In this paper, we compared various feature selection strategies with respect to their performance in selecting combinations of features that perform well in classification using multi-omics data. Feature selection is, however, only one step in the process of obtaining a strong prediction rule. The recently introduced tool BioDiscML [18] integrates feature selection and classifier selection for omics data in a fully-automated pipeline. More precisely, it combines different feature selection procedures and selects an optimal classifier out of a large pool of possible classifiers in an effort to maximize the predictive performance of the resulting biomarker signature. Continuous outcomes are supported as well.

Our study has some limitations. First, we considered only binary outcomes and it is not clear how transferable our results are to other types of outcomes, such as survival data. Second, we did not include methylation data due to their large size. However, methylation data can contain a great deal of predictive information.

## Conclusions

Feature selection has been an active and productive research area in machine learning. Its importance is unquestionable, and it has proven to be effective in improving prediction accuracy and reducing the complexity of machine learning models. Given the unique structure of multi-omics data, it is unclear how feature selection for these data should be performed. With the benchmark study presented in this paper, in which we compared eight common feature selection methods using 15 real cancer multi-omics datasets with binary outcomes, we have tried to close this gap.

Given the results of this benchmark study we recommend the embedded method RF-VI and the filter method mRMR for feature selection, where it is sufficient to use only small numbers of best features (e.g., 10). While mRMR seems to be associated with a slightly better predictive performance than RF-VI, but mRMR is computationally costly. Feature selection based on the Lasso delivers comparable or even better predictive performance, but the selected models generally have many more features than those required when using RF-VI or mRMR. Lastly, it does not seem to be necessary to perform feature selection separately for each data type. Instead, it seems to be sufficient to select the features concurrently from all data types.

## Methods

### Datasets

Herrmann et al. [19] selected cancer datasets from the TCGA (http://cancergenome.nih.gov) with more than 100 samples and five different omics blocks (mRNA, miRNA, methylation, CNV, and mutation), resulting in 26 datasets, where each contained samples from a different cancer type. Their study, similarly to our own, did not include methylation data due to their large size which would have resulted in excessive download and computational times. Therefore, for each type of cancer, there were four molecular data types accompanied by clinical data, resulting in a total of five sets of variables.

In the present paper, 11 of the 26 available datasets originally considered by Herrmann et al. were excluded. Three datasets were excluded because they did not have observations for every data type. Two datasets that did not include the outcome variable, presence of the TP53 mutation, were also excluded. Finally, five datasets with TP53 mutation rates less than 0.1 and one dataset with a mutation rate greater than 0.9 were excluded. Table 3 provides an overview of the 15 included datasets. Note that while it is not meaningful contextually to predict the presence of TP53 mutations, they have been found to be associated with poor clinical outcomes in cancer patients [20]. Against this background, we use TP53 as a surrogate for a phenotypic outcome.

**Table 3:** Summary of the datasets used for the benchmark experiment

Dataset	Cancer	Clin	cnv	mirna	mutation	rna	*f*	*n*	*m*	r_m
BLCA	Bladder urothelial	5	57,964	825	18,577	23,081	100,455	382	186	0.49
BRCA	Breast invasive C	8	57,964	835	17,975	22,694	99,479	735	255	0.35
COAD	Colon AC	7	57,964	802	18,538	22,210	99,524	191	106	0.55
ESCA	Esophageal C	6	57,964	763	12,628	25,494	96,858	106	83	0.78
HNSC	Head–neck squamous CC	11	57,964	793	17,248	21,520	97,539	443	307	0.69
LGG	Low grade glioma	10	57,964	645	9235	22,297	90,154	419	195	0.47
LIHC	Liver hepatocellular C	11	57,964	776	11,821	20,994	91,569	159	44	0.28
LUAD	Lung AC	9	57,964	799	18,388	23,681	100,844	426	212	0.50
LUSC	Lung squamous CC	9	57,964	895	18,500	23,524	100,895	418	346	0.83
PAAD	Pancreatic AC	10	57,964	612	12,392	22,348	93,329	124	78	0.63
PRAD	prostate AC	4	57,925	585	11,702	21,769	91,981	407	48	0.12
SARC	Sarcoma	11	57,964	778	10,001	22,842	91,599	126	48	0.38
SKCM	Skin cutaneous M	9	57,964	1002	18,593	22,248	99,819	249	39	0.16
STAD	Stomach AC	7	57,964	787	18,581	26,027	103,369	295	139	0.47
UCEC	Uterine corpus EC	11	57,447	866	21,053	23,978	103,358	405	144	0.36

*C*. indicates carcinoma, *AC* Adenocarcinoma, *CC* Cell carcinoma, *M* Melanoma, and *EC* Endometrial carcinoma.

The third to the seventh column show the numbers of features in the respective feature groups and the eighth column the total amount of features (*f*). The last three columns show the numbers of observations (*n*), the numbers of TP53 mutation cases (*m*), and the ratio between the numbers of mutation events and the numbers of observations (r_m), in that order

### A general overview of feature selection methods

Feature selection methods for classification can be classified in different ways. According to the relationship between feature selection and prediction, they can be classified into filter, wrapper, embedded, and hybrid methods [21–29], which is the most common classification. According to the type of the output, feature selection methods can be classified into individual evaluation and subset evaluation methods. Individual evaluation, also known as feature ranking [30], evaluates individual features by assigning weights based on their degrees of relevance. In contrast, subset evaluation generates a subset of candidate features based on a certain search strategy.

#### Filter methods

Filter algorithms carry out the feature selection process as a pre-processing step independent of the method used in the subsequent classification. They can be classified into univariate and multivariate methods. In the univariate approach, each feature is evaluated independently according to specific criteria, thus ignoring feature dependencies. Examples of these methods include infor [31] and Correlation-based Feature Selection [32]. To overcome the problem of ignoring feature dependency, multivariate feature selection methods have been proposed, for example, the mRMR method [33] and ReliefF [34].

The advantages of filter-based methods are that they are easy to implement, are expected to be faster than other types of feature selection algorithms, and are independent of the classifier. Thus, feature selection needs to be performed only once, and then different classification algorithms can be evaluated. A disadvantage of filter methods is that they ignore the interaction with the choice of the classifier.

#### Wrapper methods

The wrapper approach uses a given classifier to evaluate feature subsets and thus the feature selection process is ‘wrapped’ around the classifier [30]. In other words, the wrapper model is an iterative search process that uses the performance of the classifier at each iteration to guide the search process [35]. Wrapper methods can be classified into greedy and random search methods [14, 36]. A greedy algorithm is a simple, intuitive algorithm that makes locally optimal choices in the hope that this will lead to a globally optimal solution. It usually starts with an initial solution and updates this solution iteratively. In each iteration, some alternative solutions are generated and, based on the profitability of these solutions, the algorithm selects the best alternative solution to replace the current solution. The algorithm terminates as soon as a certain stopping criterion is fulfilled, for example, if no alternative solution would be better than the current solution or if a maximum number of iterations is reached [36, 37]. Sequential backward selection and sequential forward selection are two well-known greedy search methods [36]. The main drawback of the greedy algorithm is that large numbers of possible feature subsets must be evaluated. For high-dimensional data this number becomes too large to handle computationally. As a solution, wrapper methods based on evolutionary algorithms can be applied. These methods search the solution space randomly. Five well-known stochastic search methods are the GA [22, 38], particle swarm optimization [39, 40], bacterial foraging optimization [41], simulated annealing [42, 43], and ant colony optimization [23, 44, 45].

The main advantage of wrapper methods over filter methods is that they take the classifier into account in the feature selection. The feature subsets selected by wrapper algorithms tend to produce more accurate classifiers because the selected features are determined in such a way that they perform well when considered in combination. With filter methods we can select features that are influential, but they are less suitable for selecting combinations of features that perform well in classification. In general, "the m best features are not the best m features" [46]. A common drawback of wrapper techniques is that they have a higher risk of overfitting than filter methods if the iterative process is not stopped early and that they tend to be computationally very intensive.

#### Embedded methods

Embedded methods are feature selection mechanisms that are integral to the training process of specific prediction methods [11]. These include regularization methods and various types of decision tree algorithms. Regularization or shrinkage methods are based on a regularized model of the objective function with feature weighting to minimize the estimated generalization error while forcing the feature coefficients to be small. Some of the methods of this kind, such as Lasso [47] and elastic net [48], shrink a proportion of the coefficients exactly to zero and thus perform feature selection implicitly. An example of a decision tree-based algorithm is the RF-VI [49]. This measure ranks the features according to their importance to prediction. This ranking can be used for feature selection by selecting the features with the largest variable importance score values.

Embedded methods have the same main advantage as wrapper methods. Compared to wrapper methods, embedded methods may be less computationally intensive and less prone to overfitting. However, embedded methods often use quite strict modeling assumptions. The classification performance of embedded methods can sometimes be worse compared to filter methods and wrapper methods [50]. We did, however, not observe this in our benchmark study (see Section “Discussion”).

#### Hybrid methods

Hybrid techniques are often developed by combining two or more feature selection algorithms of different types (filter, wrapper, and embedded). They attempt to combine the benefits of these various methods into a single approach for feature selection.

For example, when dealing with high-dimensional data, the computational efficiency of a filter method is often combined with the strong predictive performance of a wrapper or embedded method to form a hybrid approach. First, a filter method is used to reduce the size of the feature space; second, a wrapper or embedded method is used to find the optimal subset of features from the retained features. For example, Akadi et al. [51] combined the mRMR method and the genetic algorithm to form a filter-wrapper hybrid method.

### Configurations of the feature selection methods compared in the benchmark study

To identify relevant feature selection methods we reviewed the overview of Momeni et al. [52], which investigated about 300 papers from Scopus in the field of feature selection and cancer classification published from 2009 to 2019, and Al-Tashi et al. [53], who surveyed multi-objective feature selection algorithms published from 2012 to 2019. We also surveyed gene selection methods used in papers on human cancer classification from PubMed published in the last 10 years. Finally, we determined the eight feature selection methods that were considered most often for cancer classification in the surveyed papers. These eight popular methods included four filter methods, two wrapper methods, and two embedded methods. An overview of all methods considered in our benchmark study is displayed in Table 4. We used R version 4.1.2 [54] in all our analyses. For all algorithms, the default parameter values in the respective R implementations were used if not indicated otherwise.

**Table 4:** Summary of methods compared in the benchmark experiment

Method	Selector	R package::function
Filter	*t*-test	::*t*.test
	Information gain (infor)	FSelector::information.gain
	ReliefF	FSelector::relief
	The Minimum Redundancy Maximum Relevance (mRMR)	mRMRe::mRMR.ensemble
Wrapper	Recursive feature elimination (Rfe)	Caret::rfeControl and rfe
	Genetic algorithm (GA)	Caret::gafsControl and gafs
Embedded	The least absolute shrinkage and selection operator (Lasso)	Glmnet::cv.glmnet
	The permutation importance of random forests (RF-VI)	Ranger:: ranger

#### Filter methods

*T*-test based feature selection [55] is a popular univariate approach. The two-sample *t*-test is a statistical test used to assess whether the means of two classes are statistically different from each other. For each feature, a *t*-test is performed and then features are ranked according to the *p-*values from these tests.

Infor [56] is an entropy-based feature evaluation method that provides an ordered ranking of the features. Entropy is a measure of the amount of information contained in a feature. When used in feature selection the information gain is the difference between the entropy of the outcome feature measured unconditionally and conditionally on an input feature. The more important the input feature is, the smaller the entropy of the outcome feature conditional on the input feature, that is, the information in the outcome feature when controlling for the input feature. Therefore, the information gain will be larger for more important input features. To use the information gain for feature selection, the input features are ranked according to their associated infor values and the largest valued features are then selected.

ReliefF [34] is a multivariate filter algorithm that is extended from the original Relief algorithm [57]. For each sample R in the dataset, the algorithm considers its k nearest neighbors from the same class as R and its k nearest neighbors from the other class. Subsequently, for each feature, the average distance of its value in R from the k nearest neighbors in the same class and the average from the k nearest neighbors in the other class are averaged. If the average distance from the opposite class is larger than that from the same class, this indicates that the feature could be useful for prediction and its weight is increased; otherwise, its weight is decreased. This is performed for all samples in the dataset and the features are ranked in descending order according to their weights.

The mRMR [33] method is a multivariate filter procedure that ranks features according to their predictive information, while accounting for their mutual information. The goal of mRMR is to retain features that have the highest relevance for predicting the target class and are also minimally redundant among each other.

#### Wrapper methods

GAs [58] are among many optimization algorithms that are inspired by nature. A simple GA starts by initializing the so-called population and subsequently runs several iterations. Each iteration consists of several steps, called GA operators: selection, crossover, and mutation. At the end of each iteration, a new generation is created as input for the next iteration. The algorithm terminates when it reaches a pre-specified number of iterations or finds the optimal solution.

Rfe [59] is a well-known iterative feature selection algorithm that employs a backward elimination method. It performs feature selection by iteratively training a classifier provided with the current set of features and discarding the least important feature as indicated by the performance of the classifier.

An important factor determining the performance of wrapper methods is when to stop the iterative process. If this process is not stopped prematurely, the resulting classifier will eventually overfit the training data, which can lead to poor performance on new data. To avoid this kind of overfitting, we used fivefold cross-validation on each training data set for determining the optimal number of iterations in the GA and the optimal number of features to retain in Rfe.

#### Embedded-based feature selector

Random forest is a tree-based ensemble method introduced by Breiman [49]. Random forest itself does not perform feature selection. RF-VI ranks the features with respect to their importance for prediction and this ranking is then used for feature selection by selecting the features with the largest variable importance scores.

Lasso [47] is a very popular embedded feature selection method due to its simplicity and effectiveness. It fits a high-dimensional regression model with a linear predictor and applies L1-regularization to penalize large feature coefficient values. In the process of shrinking the coefficients, numerous coefficients are set to zero. The features with non-zero coefficients are considered relevant.

### Experimental settings

We varied five parameters in our analyses:

- Feature selection method: eight methods were compared, see Table 4; according to their relationship with the classification method: four filter methods, two wrapper methods, and two embedded methods; according to the type of the output: five rank methods and three subset evaluation methods.
- The number of selected features: for the rank methods, the number of selected features (nvar) was set to 10, 100, 1000, and 5000. An alternative would have been to establish thresholds in the values of the feature importance scores. However, determining where to establish such thresholds is not an easy problem to solve. Moreover, the different numbers of selected features considered in our study correspond to different types of prediction rules. For example, the choice 10 corresponds to prediction rules based on only a few markers, while the choice 5000 corresponds to high-dimensional prediction rules that take large numbers of features into account. For the subset evaluation methods, the numbers of selected features were determined by the optimized feature subsets.
- Feature selection type: separate selection and selection from all blocks at the same time (non-separate selection). For separate selection, in the case of the rank methods, the numbers of selected features per data type were set proportional to the total of the numbers of features in all data types.
- Clinical variables: including versus excluding clinical data.
- Classification method: support vector machine, random forests.

For both considered classification methods, we considered all possible combinations of these parameter values, and there are 16 settings for the rank methods (4 × 2 × 2) and four settings for the subset evaluation methods (2 × 2).

In the cases of the wrapper methods and the embedded methods, the computation time becomes very large if the number of features is large, which is the case for multi-omics data. Therefore, before applying these methods, we used *t*-test based filtering to select the top 10% of features to reduce the computational consumption.

The accuracy, the AUC, and the Brier were used to evaluate the predictive performance. As an evaluation scheme, we used fivefold cross-validation repeated three times to measure the performance of each method on each dataset.

For each setting, we tested for differences between the dataset-specific performance measure values obtained with the different methods using the Friedman test. Applying the Holm-Bonferroni procedure [60], we adjusted the resulting *p-*values for multiple testing, separately for the two considered classification methods and for rank methods and subset evaluation methods, respectively. Note that, while the performance measure values obtained for the cross-validation iterations based on the same datasets are not independent, the mean performance measure values per dataset are independent of one another. Thus, the assumption of independence of the observations that is underlying the Friedman test is not violated.

## Supplementary Information

**Additional file 1: Figures S1a and S2a** show the distributions of the mean cross-validated accuracy values across the datasets for all rank-methods using the RF and SVM classifiers, respectively. **Figs. S1b and S2b** show the mean cross-validated accuracy values obtained for all subset evaluation methods using the RF and SVM classifiers, respectively. **Figs. S3a and S4a** show the distributions of the mean cross-validated Brier score across the datasets for all rank methods using the RF and SVM classifiers, respectively. **Fig. S3b and S4b** show the mean cross-validated Brier score obtained for all subset evaluation methods using the RF and SVM classifiers, respectively. **Table S1:** The best performing methods (according to the AUC) per setting for SVM. **Table S2:** The best performing methods and settings (according to the AUC) per dataset for SVM

## Data Availability

All R code written to produce and evaluate our results is available on GitHub (https://github.com/yingxiali/feature-selection, accessed on March 22, 2022). Moreover, the pre-processed data sets are available as CSV files on the online open access repository figshare (https://doi.org/10.6084/m9.figshare.20060201.v1).

## Abbreviations

TCGA: The cancer genome atlas
AUC: The area under the receiver operating characteristic curve
Brier: Brier score
SVM: Support vector machine
RF: Random forests
infor: Information gain
mRMR: The minimum redundancy maximum relevance
Rfe: Recursive feature elimination
GA: Genetic algorithm
Lasso: The least absolute shrinkage and selection operator
RF-VI: The permutation importance of random forests
