# Supplementary material for: Benchmark study of feature selection strategies for multi-omics data
Source: BMC Bioinformatics. 2022 Oct 5;23:412. doi: 10.1186/s12859-022-04962-x (PMC9533501; doi:10.1186/s12859-022-04962-x)
Supplement: Supplementary file 1 — Additional file 1: Figures S1a and S2a show the distributions of the mean cross-validated accuracy values across the datasets for all rank-methods using the RF and SVM classifiers, respectively. Figs. S1b and S2b show the mean cross-validated accuracy values obtained for all subset evaluation methods using the RF and SVM classifiers, respectively. Figs. S3a and S4a show the distributions of the mean cross-validated Brier score across the datasets for all rank methods using the RF and SVM classifiers, respectively. Fig. S3b and S4b show the mean cross-validated Brier score obtained for all subset evaluation methods using the RF and SVM classifiers, respectively. Table S1: The best performing methods (according to the AUC) per setting for SVM. Table S2: The best performing methods and settings (according to the AUC) per dataset for SVM [file 12859_2022_4962_MOESM1_ESM.docx]

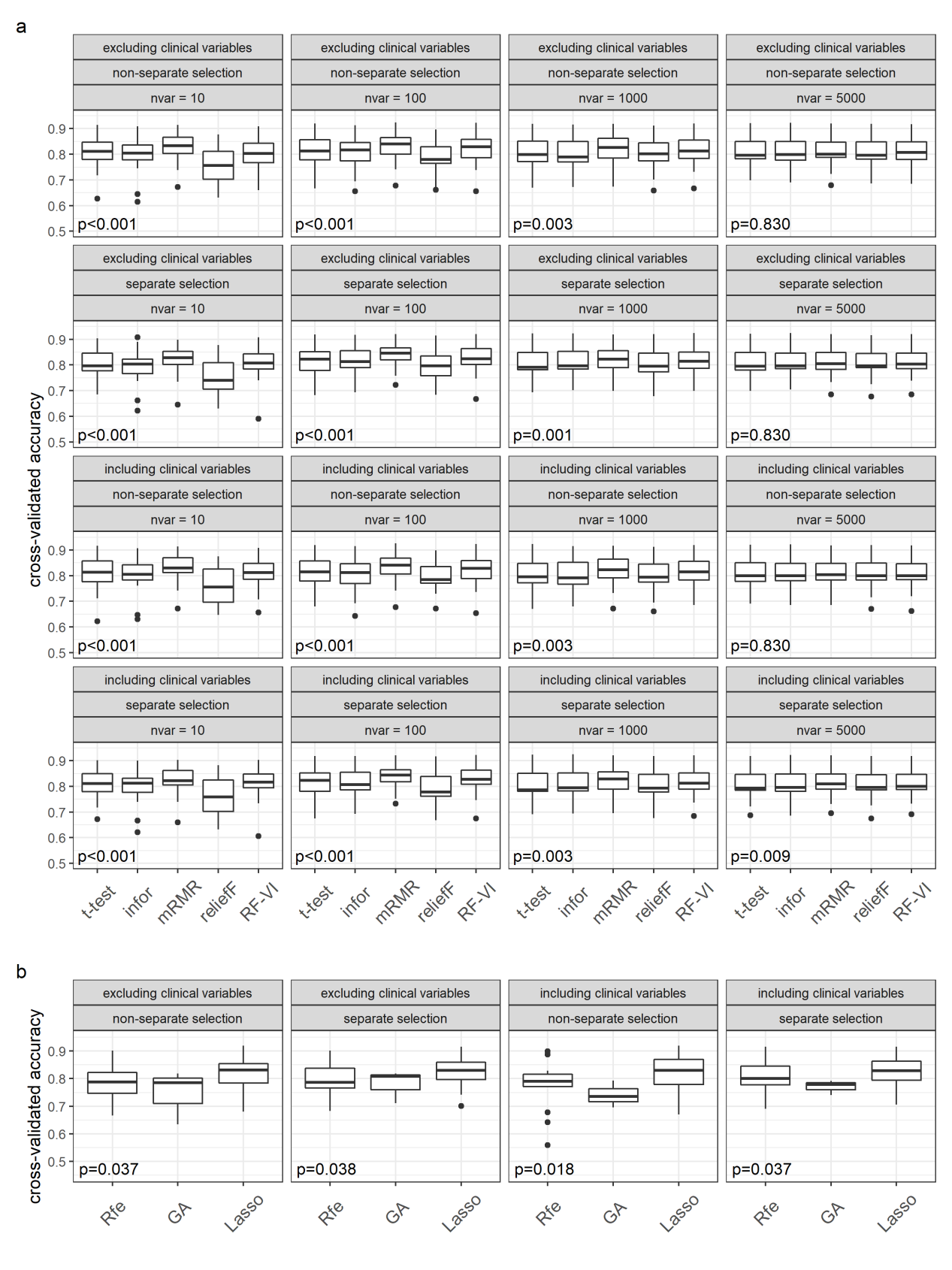


Figure S1: Prediction performance using RF after feature selection. Panels a and b show the distributions of the mean cross-validated accuracy values across the datasets for all rank and subset evaluation methods, respectively. The p-values show the results of the Friedman tests.


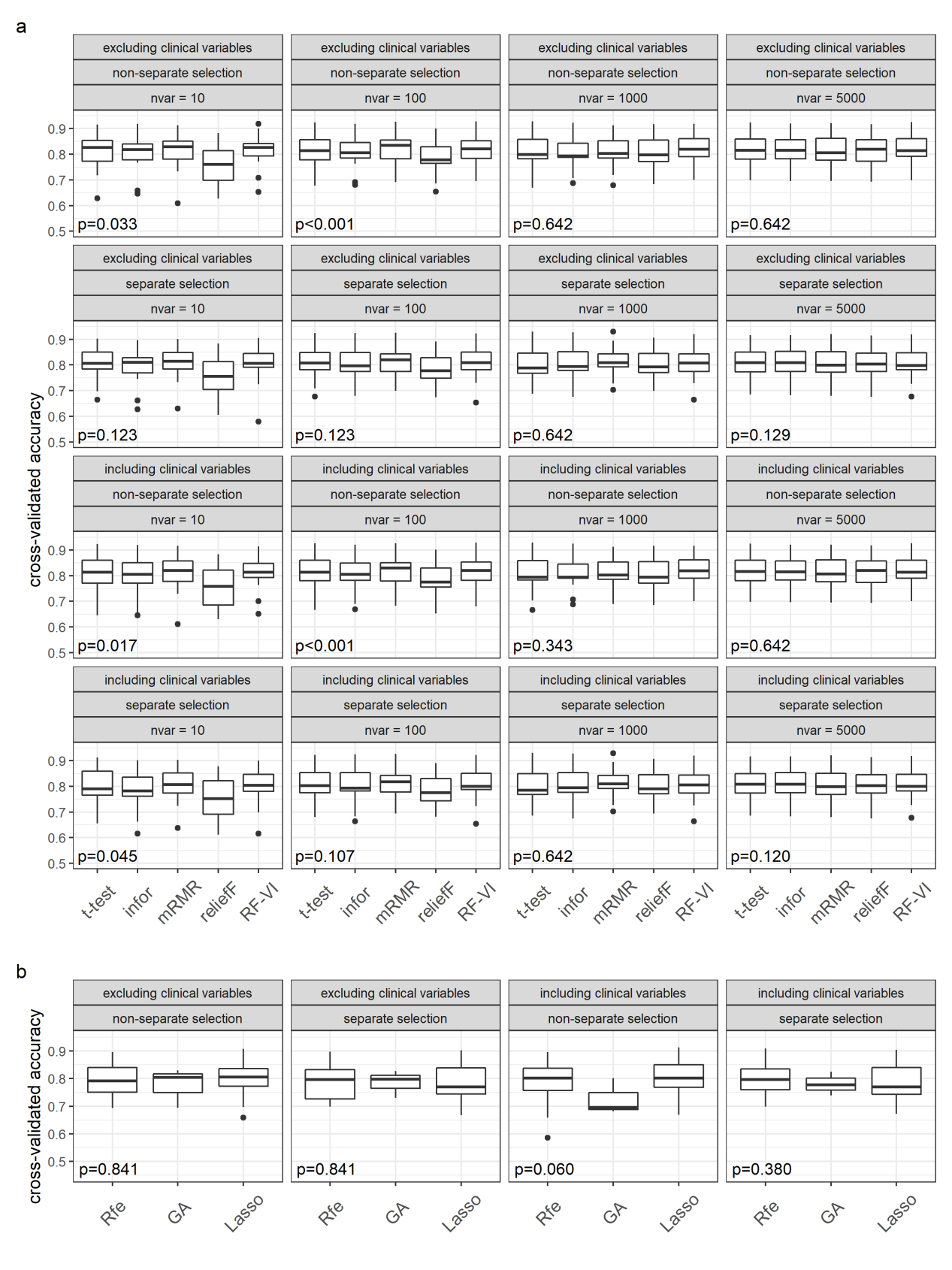


Figure S2: Prediction performance using SVM after feature selection. Panels a and b show the distributions of the mean cross-validated accuracy values across the datasets for all rank and subset evaluation methods, respectively. The p-values show the results of the Friedman tests.


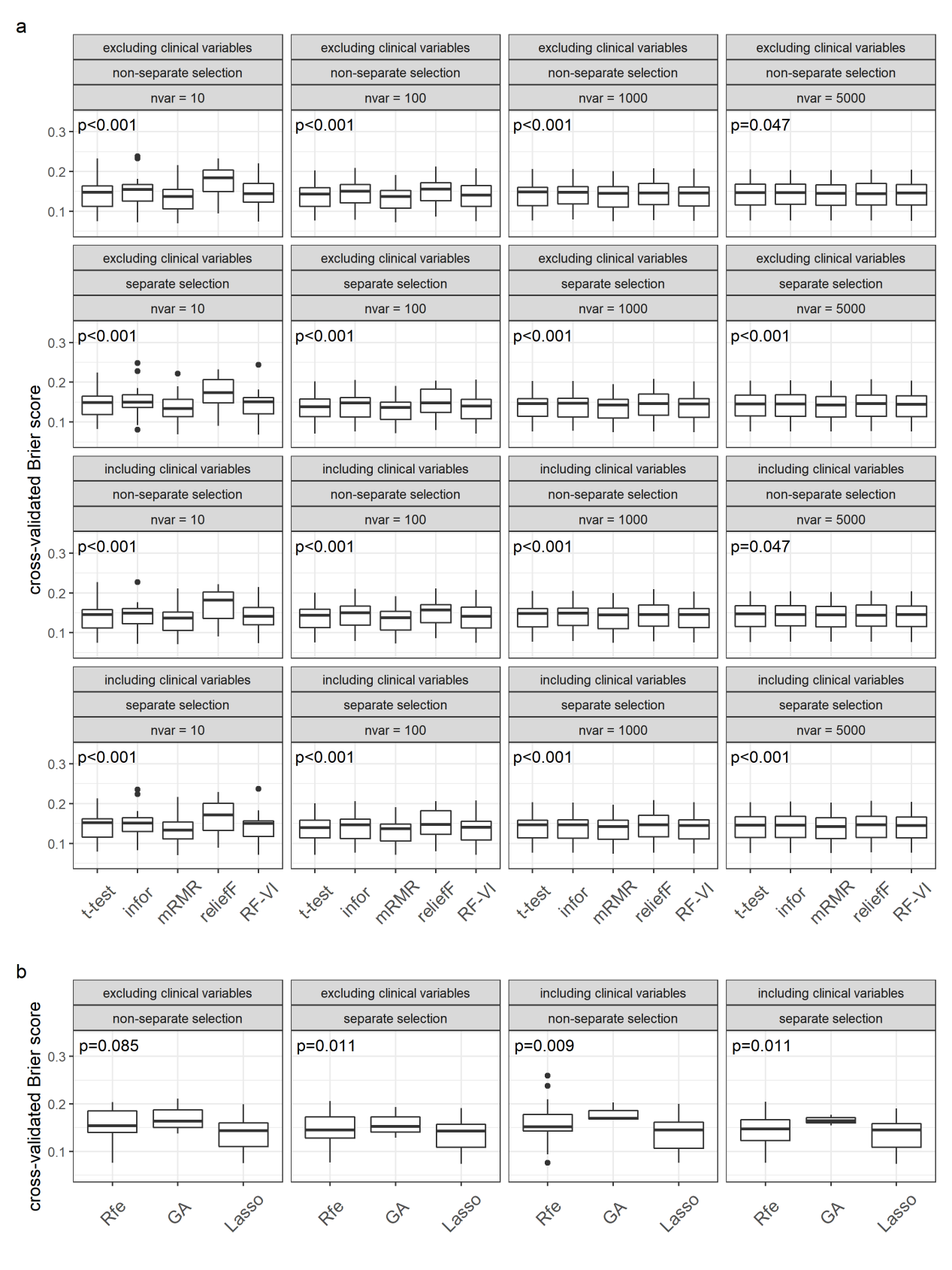


Figure S3: Prediction performance using RF after feature selection. Panels a and b show the distributions of the mean cross-validated Brier score values across the datasets for all rank and subset evaluation methods, respectively. The p-values show the results of the Friedman tests.


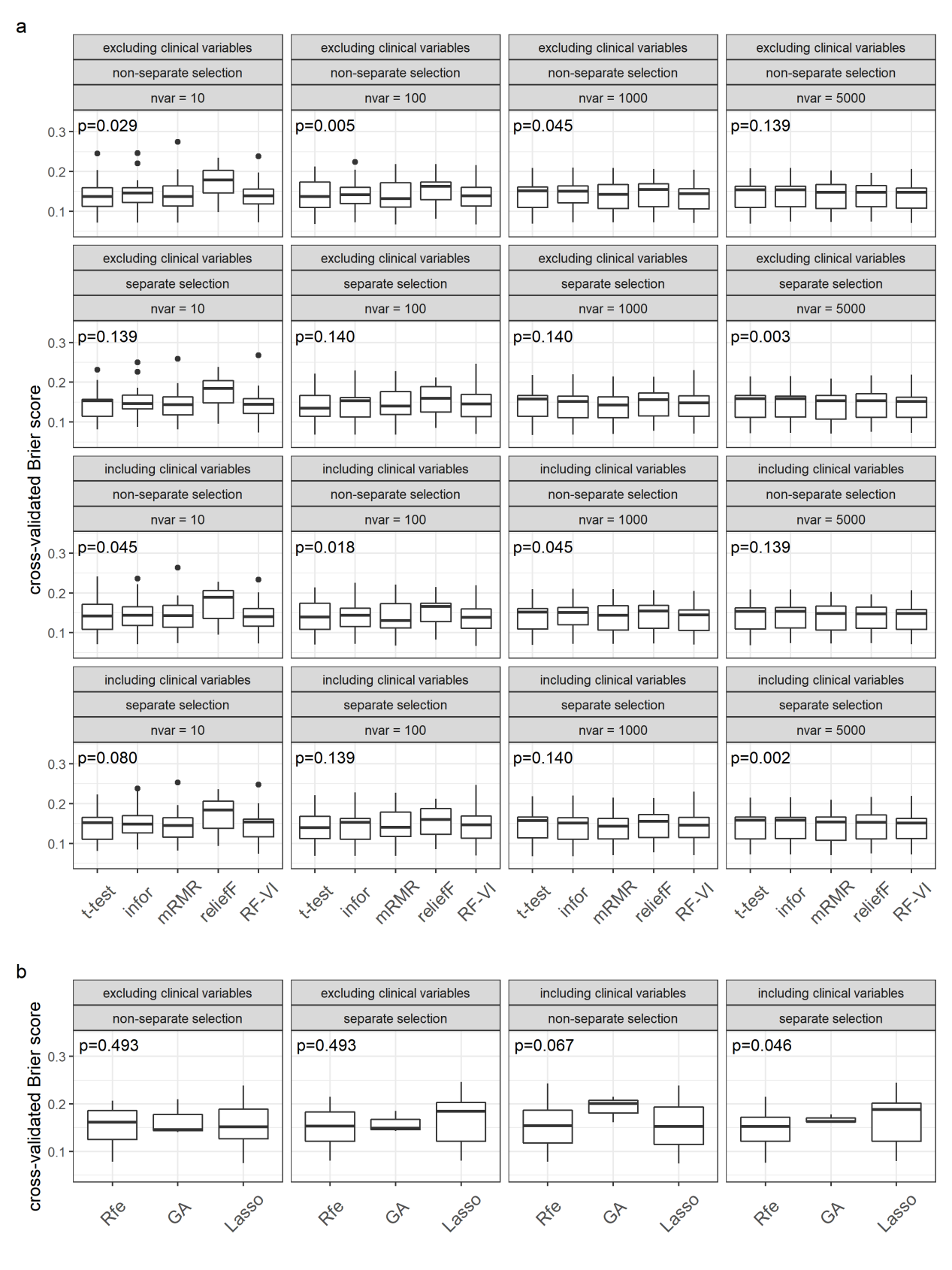


Figure S4: Prediction performance using SVM after feature selection. Panels a and b show the distributions of the mean cross-validated Brier score values across the datasets for all rank and subset evaluation methods, respectively. The p-values show the results of the Friedman tests.

Table S1. The best performing methods (according to the AUC) per setting for SVM. The values of the performance metrics were obtained by averaging over the cross-validation repetitions and datasets; ‘nvar’ denotes the number of selected features, ‘selsep’ whether the features were selected separately by data type, and ‘clivar’ whether clinical variables were included or not.

| nvar | selsep | clivar | selector | auc | brier | accuracy |
| --- | --- | --- | --- | --- | --- | --- |
| 10 | no | no | mRMR | 0.8110 | 0.1432 | 0.8115 |
| 10 | no | yes | mRMR | 0.8171 | 0.1436 | 0.8105 |
| 10 | yes | no | mRMR | 0.8072 | 0.1470 | 0.8074 |
| 10 | yes | yes | mRMR | 0.8101 | 0.1477 | 0.8038 |
| 100 | no | no | RF-VI | 0.8280 | 0.1358 | 0.8202 |
| 100 | no | yes | RF-VI | 0.8292 | 0.1359 | 0.8190 |
| 100 | yes | no | t-test | 0.8248 | 0.1404 | 0.8101 |
| 100 | yes | yes | t-test | 0.8235 | 0.1409 | 0.8097 |
| 1000 | no | no | RF-VI | 0.8359 | 0.1351 | 0.8196 |
| 1000 | no | yes | RF-VI | 0.8361 | 0.1351 | 0.8196 |
| 1000 | yes | no | mRMR | 0.8335 | 0.1388 | 0.8164 |
| 1000 | yes | yes | mRMR | 0.8333 | 0.1389 | 0.8157 |
| 5000 | no | no | RF-VI | 0.8284 | 0.1377 | 0.8162 |
| 5000 | no | yes | mRMR | 0.8283 | 0.1393 | 0.8130 |
| 5000 | yes | no | mRMR | 0.8268 | 0.1398 | 0.8088 |
| 5000 | yes | yes | mRMR | 0.8267 | 0.1399 | 0.8087 |
| - | no | no | Lasso | 0.8085 | 0.1577 | 0.8005 |
| - | no | yes | Lasso | 0.8106 | 0.1570 | 0.8018 |
| - | yes | no | RF-VIe | 0.8097 | 0.1491 | 0.7925 |
| - | yes | yes | RF-VIe | 0.8183 | 0.1463 | 0.8002 |

Table S2. The best performing methods and settings (according to the AUC) per dataset for SVM. Here, ‘nvar’ denotes the number of selected features, ‘selsep’ whether the features were selected separately by data type, and ‘clivar’ whether clinical variables were included or not.

| dat | selector | nvar | selsep | clivar |
| --- | --- | --- | --- | --- |
| BLCA | t-test | 100 | no | no |
| BRCA | mRMR | 10 | no | yes |
| COAD | t-test | 100 | no | no |
| ESCA | reliefF | 5000 | no | no |
| HNSC | Lasso | - | yes | yes |
| LGG | RF-VI | 1000 | yes | no |
| LIHC | mRMR | 5000 | yes | no |
| LUAD | t-test | 100 | no | no |
| LUSC | RF-VIe | - | no | no |
| PAAD | infor | 1000 | no | yes |
| PRAD | RF-VI | 1000 | yes | no |
| SARC | GA | - | yes | yes |
| SKCM | t-test | 10 | no | yes |
| STAD | RF-VI | 1000 | yes | no |
| UCEC | Lasso | - | yes | no |
